# Supplementary material for: Odor Profile of Four Cultivated and Freeze-Dried Edible Mushrooms by Using Sensory Panel, Electronic Nose and GC-MS
Source: J Fungi (Basel). 2022 Sep 11;8(9):953. doi: 10.3390/jof8090953 (PMC9504341; doi:10.3390/jof8090953)
Supplement: Supplementary file 1 [file jof-08-00953-s001.zip › jof-1896215-supplementary.pdf]

SUPPLEMENTARY MATERIAL

Article

Odor Profile of Four Cultivated and Freeze-dried Edible Mushrooms by Using Sensory Panel, Electronic Nose and GC-MS

Inmaculada Gómez [1]; Rebeca Lavega González [2]; Eva Tejedor-Calvo [3,4]; Margarita Pérez Clavijo [2]; Jaime Carrasco [2,5,*]

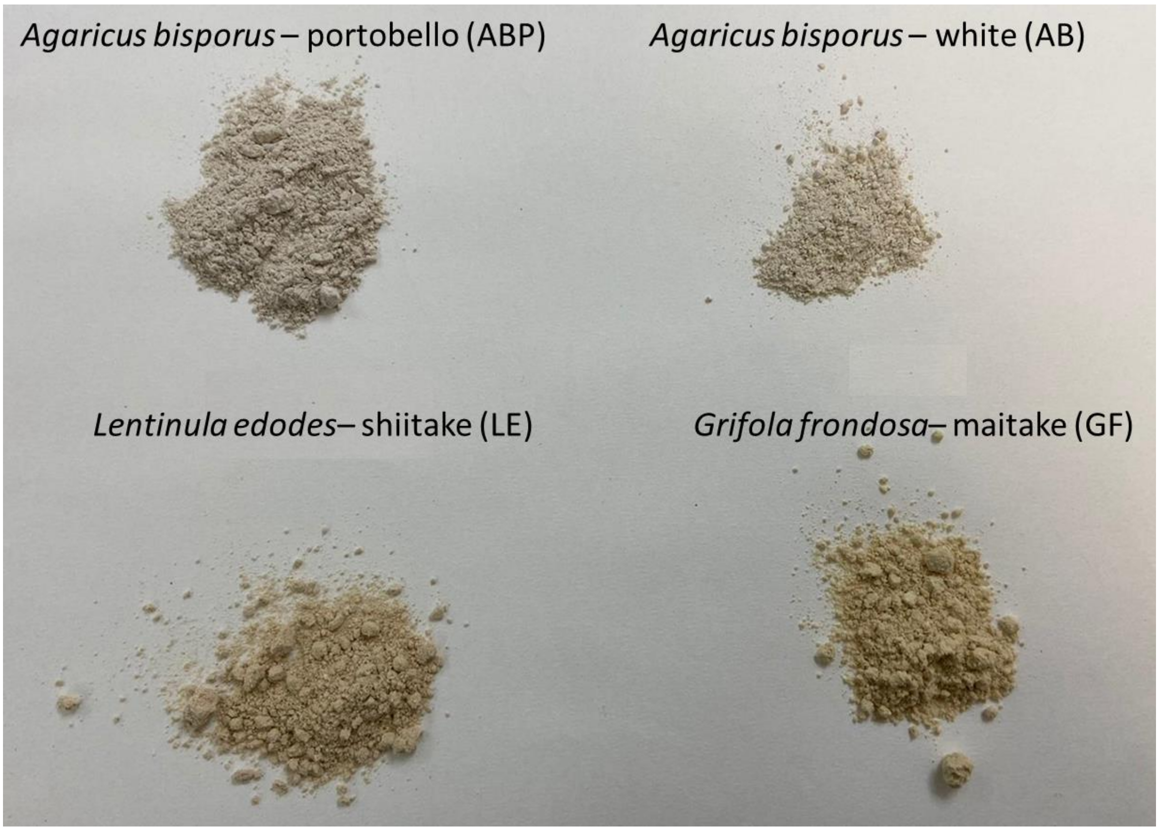

Figure S1. Freeze-dried samples obtained from cultivated mushroom and employed in the analysis.

Table S1. Percentage of the most abundant VOCs detected in the freeze-dried samples by the optimised method based in the SPME fibre extraction and GC-MS analysis.

**VOC**[1]	**CAS**[2]	**ABP**[3]	**AB**	**LE**	**GF**
1-butanol,2-methyl	137-32-6	0.50	1.04	2.34	0.71
1-butanol,3-methyl	123-51-3	0.58	0.51	3.25	3.43
1-dodecene	112-41-4	0.58	0.69	*n.d.	n.d.
1-hexenol	111-27-3	27.71	23.33	7.93	3.48
1-octen-3-ol	3391-86-4	6.30	6.76	2.27	4.40
1-octen-3-one	4312-99-6	3.28	1.42	1.46	3.31
1-pentanol	71-41-0	3.62	3.81	3.78	0.87
2,3-butadienol	513-85-9	n.d.	n.d.	6.12	n.d.
2-heptanone	110-43-0	2.43	2.50	1.76	2.86
2-octenal-2-butyl	13019-16-4	0.58	0.53	1.17	0.88
2-pentylfurane	3777-69-3	n.d.	4.86	1.14	7.40
2-propanone	67-64-1	2.07	3.22	1.52	1.32
3-octanol	598-98-0	2.82	5.30	n.d.	6.73
3-octanone	106-68-3	10.99	14.72	n.d.	16.35
3-octen-2-one	1669-44-9	3.94	n.d.	1.55	3.08
4-methyloctane	2216-34-4	1.58	3.06	3.82	0.79
benzaldehyde	100-52-7	11.96	9.34	0.81	2.88
benzeneacetaldehyde	122-78-1	n.d.	n.d.	0.56	0.75
butanal-2-methyl	96-17-3	1.30	1.30	4.75	6.17
butanal-3-methyl	590-86-3	1.84	1.69	13.85	0.15
dimethylsulfone	67-71-0	0.13	0.13	1.01	0.80
dodecane	112-40-3	1.21	1.58	0.79	1.84
gamma-caprolactone (=*gamma*-hexalactone)	695-06-07	n.d.	n.d.	4.73	4.31
gamma-nonalactone	104-61-0	0.18	0.23	5.30	0.90
gamma-valerolactone (=*gamma*-pentalactone)	108-29-2	n.d.	n.d.	2.84	2.07
hexanal	66-25-1	12.41	11.42	11.51	15.63
l-camphor	76-22-2	n.d.	n.d.	1.17	n.d.
limonene	138-86-3	0.71	0.87	4.27	1.17
linalool	78-70-6	n.d.	n.d.	n.d.	5.44
octane	111-65-9	n.d.	n.d.	2.62	n.d.
pentanal	110-62-3	2.57	1.71	3.64	2.27
propanol	71-23-8	0.70	n.d.	4.03	n.d.

[1]VOC: Volatile Organic Compound; [2]CAS: Number assigned to every volatile compound (chemical) detected and quantified; [3]ABP: *Agaricus bisporus* spp. *brunnescens*; AB: *Agaricus bisporus* spp. *bisporus*; LE: *Lentinula edodes*; GF: *Grifola frondosa*; *n.d.: not detected.
